# Identification of *ATP2B4* Regulatory Element Containing Functional Genetic Variants Associated with Severe Malaria

**DOI:** 10.3390/ijms23094849

**Published:** 2022-04-27

**Authors:** Samia Nisar, Magali Torres, Alassane Thiam, Bruno Pouvelle, Florian Rosier, Frederic Gallardo, Oumar Ka, Babacar Mbengue, Rokhaya Ndiaye Diallo, Laura Brosseau, Salvatore Spicuglia, Alioune Dieye, Sandrine Marquet, Pascal Rihet

**Affiliations:** 1 MarMara Institute, Aix-Marseille University INSERM, TAGC, UMR_S_1090, 13288 Marseille, France; samia.biotech@yahoo.com (S.N.); magali.torres@univ-amu.f (M.T.); bruno.pouvelle@univ-amu.fr (B.P.); florian.rosier91@gmail.com (F.R.); frederic.gallardo@inserm.fr (F.G.); laura_brosseau@hotmail.fr (L.B.); salvatore.spicuglia@inserm.fr (S.S.); 2 Unité d’Immunogénétique, Institut Pasteur de Dakar, Dakar BP220, Senegal; alassane.thiam@pasteur.sn (A.T.); alioune.dieye@ucad.edu.sn (A.D.); 3 Service d’Immunologie, Université Cheikh Anta Diop de Dakar, Dakar BP5005, Senegal; oumarka74@gmail.com (O.K.); babacar.mbengue@ucad.edu.sn (B.M.); 4 Service de Génétique Humaine, Faculté de Médecine, de Pharmacie et d’Odontologie, UCAD, Dakar BP5005, Senegal; rokhaya9.ndiaye@ucad.edu.sn

**Keywords:** regulatory element, enhancer, promoter, SNP, functional genomics, gene reporter, CRISPR-cas9, *ATP2B4*, calcium, malaria

## Abstract

Genome-wide association studies for severe malaria (SM) have identified 30 genetic variants mostly located in non-coding regions. Here, we aimed to identify potential causal genetic variants located in these loci and demonstrate their functional activity. We systematically investigated the regulatory effect of the SNPs in linkage disequilibrium (LD) with the malaria-associated genetic variants. Annotating and prioritizing genetic variants led to the identification of a regulatory region containing five *ATP2B4* SNPs in LD with rs10900585. We found significant associations between SM and rs10900585 and our candidate SNPs (rs11240734, rs1541252, rs1541253, rs1541254, and rs1541255) in a Senegalese population. Then, we demonstrated that both individual SNPs and the combination of SNPs had regulatory effects. Moreover, CRISPR/Cas9-mediated deletion of this region decreased *ATP2B4* transcript and protein levels and increased Ca^2+^ intracellular concentration in the K562 cell line. Our data demonstrate that severe malaria-associated genetic variants alter the expression of *ATP2B4* encoding a plasma membrane calcium-transporting ATPase 4 (PMCA4) expressed on red blood cells. Altering the activity of this regulatory element affects the risk of SM, likely through calcium concentration effect on parasitaemia.

## 1. Introduction

Malaria caused by *Plasmodium falciparum* parasites is a major cause of morbidity and mortality in many developing countries, predominantly in endemic areas of Sub-Saharan Africa. The disease outcome is variable and may be subjective to a combination of various factors, including host genetic factors and parasite virulence, in addition to environmental factors [1,2]. *P. falciparum* infection causes various clinical phenotypes, from asymptomatic parasitemia and uncomplicated malaria to severe malaria (SM) [3].

There is a growing body of evidence that human genetic factors influence the outcome of infection since the discovery of the protective effect of the sickle cell mutant (rs334). Heritability studies reported that genetic factors explain 20% to 25% of the variations in malaria phenotypes [1,4,5,6]. Genetic linkage analyses conducted in Africa provided evidence of linkage of uncomplicated malaria with chromosome 6p21 [7,8,9], whereas parasitemia was genetically linked to chromosome 5q31-q33 [6,7,10,11,12,13]. Moreover, several GWAS of SM have identified malaria resistance loci [14,15,16,17,18,19]. However, a limited number of loci and genetic variants were identified and replicated in GWAS, which were as follows: *ATP2B4* on chromosome 1, *FREM3-GYP A/B* on chromosome 4, *EPHA7* on chromosome 6, *ABO* on chromosome 9, and *HBB* on chromosome 11. rs334 (a nonsense polymorphism) is the main causal SNP within the *HBB* locus [20], and a copy number variant within *GYP A/B* locus was proven to provide protection against malaria in East Africa [21]. The specific variant DUP4 encodes hybrid glycophorin proteins that may alter the invasion of red blood cells by the parasite [22,23]. Moreover, it is thought that regulatory genetic variants alter SM resistance. Lessard et al. (2017) evidenced that an enhancer containing rs10751450, rs10751451, and rs10751452 influences the expression of *ATP2B4*, which encodes for PMCA4 (Plasma membrane calcium transporting ATPase4), the major calcium pump of red blood cells [24]. Identifying causal variants from GWAS results remains challenging, although some important results have been obtained. It is thought that unknown non-coding variants may alter the expression of genes associated with malaria phenotypes.

Here we looked for regulatory SNPs in linkage disequilibrium (LD) with tagSNPs, for which association with SM has been replicated in independent populations. We prioritized and annotated the SNPs and further investigated the best candidates for their functional activity. We provide evidence that several SNPs were associated with SM in a Senegalese population, alter the activity of an Epromoter affecting the expression of *ATP2B4* and modulate intracellular calcium concentration.

## 2. Results

### 2.1. Prioritization and Annotation of Putative Regulatory SNPs in Linkage Disequilibrium with tagSNPs Associated with Severe Malaria

We focused our analysis on the SNPs for which significant GWAS signals have been replicated: rs4951377 and rs10900585 at the *ATP2B4* locus, rs186873296 at the *FREM3-GYP A/B* locus, rs62418762 at the *EPHA7* locus, and rs8176719 at the *ABO* locus. We excluded the coding variation rs334, which captured the GWAS signal at the *HBB* locus. We identified 126 SNPs in LD with the malaria-associated SNPs, based on an r^2^ higher than 0.6. We excluded rs181620317, which is a missense variant within the *FREM3* coding sequence, and further prioritized the SNPs for their potential regulatory effect using IW scoring [25]. Moreover, we investigated the ability of sequences containing the SNPs to bind transcription factors using the ReMap tool [26], and we determined whether the 125 SNPs have been annotated as eQTLs or identified as regulatory SNPs [27]. (Table 1; Supplementary Table S1). 

**Figure 1:**
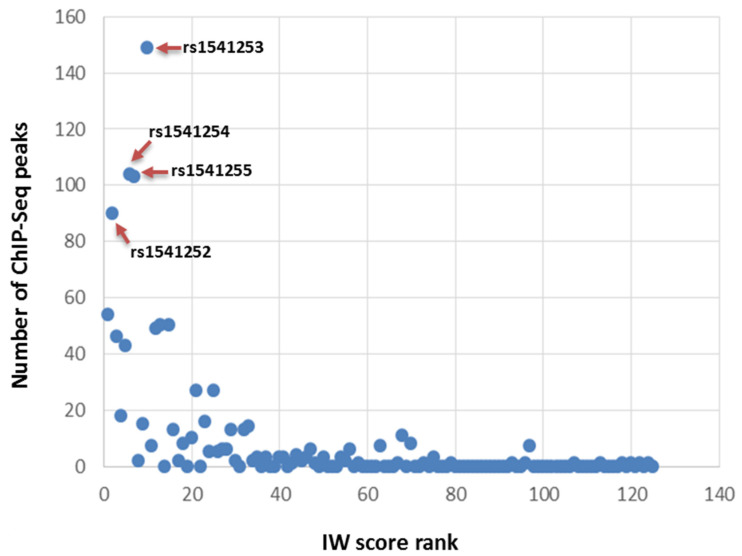
Plot displaying the integrated results for the prioritization of best candidate SNPs. IW scoring method prioritizes functionally relevant noncoding variants. The graph displays the IW score ranks of 125 candidate SNPs. The number of ChIP-seq peaks was extracted from ReMap, which integrates the results of transcription factor ChIP-seq experiments. The graph displays the number of DNA-binding protein ChIP-seq peaks for each candidate SNP. The SNPs with the best IW score rank had the highest number of ChIP-seq peaks.

**Figure 2:**
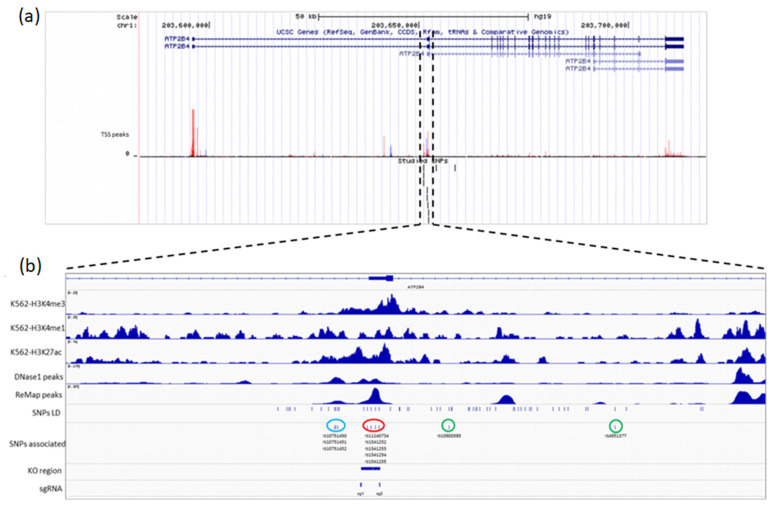
Visual representation of the *ATP2B4* locus and the epigenomic marks of the region containing the five *ATP2B4* candidate variants. (**a**) Transcripts, TSS peaks, and studied SNPs are displayed. The two long transcripts (*ATP2B4*-203 and *ATP2B4*-204) and the short transcripts are displayed. The main TSS peaks are visualized. (**b**) The candidate SNPs (rs11240734, rs1541252, rs1541253, rs1541254, and rs1541255; encircled red) are on chromosome 1, located within a DNaseI hypersensitivity region and peaks of H3K4me3, H3K4me1, and H3K27ac histone marks. The two tagSNPs (rs10900585 and rs4951377; encircled green) are located neither in peaks of ChIP-seq in the ReMap catalog nor in other epigenomic marks. The three additional regulatory variants (rs10751450, rs10751451, and rs10751452; encircled blue), previously identified as functional SNPs, are displayed.

As displayed in Figure 1, the number of transcription factor peaks identified by ChIP-seq negatively correlated with the rank of the corresponding SNPs (rho = −0.652, *p* = 2.17·10^−11^). The highest numbers of peaks were observed for rs1541252, rs1541253, rs1541254, and rs1541255 (*n* > 90), which were ranked second, 10th, 6th, and 7th based on their IW scores, respectively (Table 1). The corresponding transcription factors are displayed in Supplementary Table S2; these include GATA1, RELA, GABPA, TCF3, ELF1, NFRKB, and RUNX1. The best SNP according to the IW scoring method was rs11240734, for which 54 peaks of ChIP-seq were registered in the ReMap catalog. Strikingly, those 5 SNPs are close to each other on chromosome 1 in the *ATP2B4* region. These SNPs are located within a DNAse I hypersensitivity region and peaks of H3K4me1, H3K4me3, and H3K27ac marks in the K562 cell line, as displayed in Figure 2. Similar epigenomic marks have been found at these positions in erythroblasts (Supplementary Figure S1). rs11240734 and rs1541252 have been annotated as eQTLs, according to Haploreg, whereas rs1541255 was found to have a regulatory effect according to the Sure-seq method [28].

The prioritization was based on IW scoring analysis on the X-axis and the number of ChIP-seq peaks on the Y-axis. The four SNPs rs1541252, rs1541253, rs1541254, and rs1541255 have the highest number of ChIP-seq peaks (*n* > 90) as displayed. These SNPs are among the top 10 SNPs according to IW scoring.

Interestingly, the TaqSNPs rs4951377 and rs10900585 that are in LD with rs11240734, rs1541252, rs1541253, rs1541254, and rs1541255 had a low IW score and were ranked at positions 51 and 60, respectively (Supplementary Table S1). Furthermore, there were neither peaks of ChIP-seq in the ReMap catalog nor peaks of H3K4me1, H3K4me3, and H3K27ac marks in the K562 cell line for both SNPs (Figure 2), suggesting that they have no functional role. In contrast, rs10751450, ranked 3rd with 46 peaks of Chip-Seq, is located within a DNAse I hypersensitivity region, and peaks of H3K4me1 and H3K27ac marks in the K562 cell line (Figure 2), are located within a regulatory region [24]. Similar epigenomic profiles were observed in erythroblasts (Supplementary Figure S1). The SNPs rs10751451 and rs10751452, close to the rs10751450 [24], were ranked 12th and 13th, and rs10751451 was annotated as an eQTL according to Haploreg (Table 1). Figure 2 illustrates the position of the region containing rs10751450, rs10751451, and rs10751452 and the one containing rs11240734, rs1541252, rs1541253, rs1541254, and rs1541255. Overall, SNP prioritization and annotation allowed us to identify 5 new SNPs with potential regulatory effects, which may drive the observed association of their tagSNPs with malaria susceptibility.

### 2.2. Association between the ATP2B4 tagSNP rs10900585 and Severe Malaria in a Senegalese Cohort

One hundred and ninety-six individuals from the Senegalese cohort were successfully genotyped by sequencing. The frequencies of observed genotypes conformed to the Hardy–Weinberg equilibrium (*p* = 0.52) (Supplementary Table S3) and the allelic frequencies for rs10900585-T and G were found to be 0.59 and 0.41, respectively. We detected a significant association (*p* = 0.029; OR = 1.94) of *ATP2B4* rs10900585 with SM (Table 2) without the effect of any covariate. The TT genotype that was more frequent among cases (48.7%) than controls (32.9%) was identified as the risk genotype. Nevertheless, there was no significant association when considering age as a covariate (*p* = 0.055) (Supplementary Table S4). 

**Figure 3:**
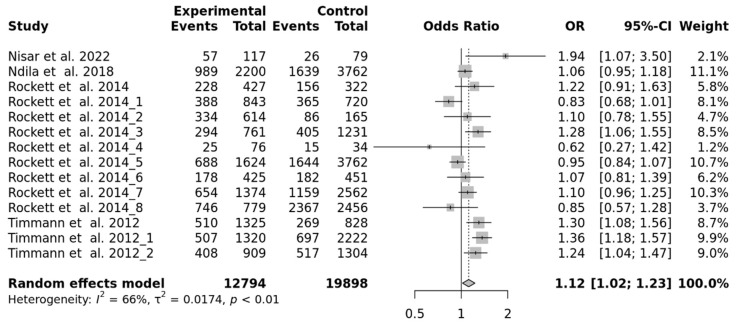
Forest plot displaying meta-analysis for the association between rs10900585 and SM.

We added 13 independent populations to our study population for performing a meta-analysis, including 12,794 SM cases and 19,898 controls (Supplementary Table S5) [19,29,30]. There was no deviation from the Hardy–Weinberg equilibrium (*p* > 0.05) but there was a significant heterogeneity among the studies (I^2^ = 66%; tau^2^ = 0.0174; *p* = 0.0003). Thus, the random effect model was used for calculating the combined odd ratio and 95% confidence interval (CI). There was an association of rs10900585 with SM under the genetic dominant model (OR = 1.12; 95% CI = 1.02–1.23) (Figure 3). 

### 2.3. Association of rs11240734, rs1541252, rs1541253, rs1541254, and rs1541255 with Severe Malaria in Senegalese Population

**Figure 4:**
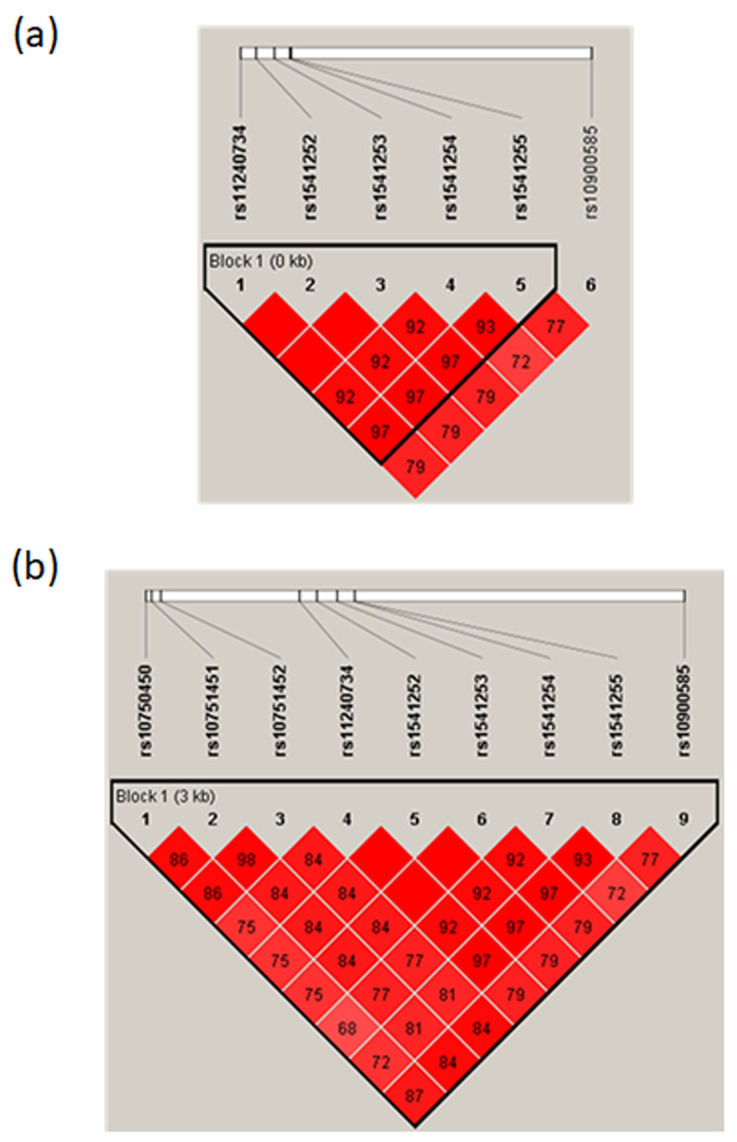
Linkage disequilibrium (LD) (r-squared) plot for the *ATP2B4* variants in the Senegalese population. (**a**) LD between the candidate five SNPs and the tagSNP rs10900585 (**b**) LD between the eight candidate SNPs and the tagSNP rs10900585 displaying r^2^ from 0.72–1.

These five SNPs newly identified as potential regulatory variants were in a 600 bp DNA fragment that we sequenced to determine the genotypes of the subjects. None of the polymorphisms deviated from the Hardy–Weinberg equilibrium (Supplementary Table S3). We compared subjects carrying the homozygous genotype for the major alleles with those carrying either the heterozygous genotype or the homozygous genotype for the minor alleles. All these SNPs were significantly associated with the disease (*p* value = 0.002), with an estimated OR of 2.52 (1.39–4.58) (Table 2), indicating a stronger association compared to the tagSNP. Among the SM individuals, 53.8% carried the rs11240734TT, the rs1541252CC, the rs1541253CC, the rs1541254GG, and the rs1541255AA genotypes, whereas only 31.6% of the control individuals carried these genotypes. The association of the five SNPs with SM remained significant (*p* = 0.006) after considering age as a covariate (Supplementary Table S4). In addition, we found a strong LD between all five SNPs in addition to the tagSNP with an r^2^ varying from 0.72 to 1 (Figure 4a). 

### 2.4. Association of rs10751450, rs10751451, and rs10751452 with Severe Malaria in Senegalese Population

Although suggested as regulatory polymorphisms [24], the SNPs rs10751450, rs10751451, and rs10751452 have never been genotyped and tested for their association with malaria. We tested whether these three SNPs were associated with SM. The frequencies of observed genotypes conformed to the Hardy–Weinberg equilibrium (Supplementary Table S3). The analysis indicated a significant association between malaria disease and these SNPs, rs10751450 (*p* = 0.016; OR 2.07), rs10751451 (*p* = 0.002; OR 2.49) and rs10751452 (*p* = 0.006; OR 2.27) (Table 2), which remained significant when considering age as a covariate (Supplementary Table S4). Noticeably, in our study population, these SNPs were found to be in strong LD between them in addition to our five new candidate SNPs (Figure 4b).

### 2.5. Haplotype Analysis of the ATP2B4 SNPs 

Haplotype analysis, combining rs11240734, rs1541252, rs1541253, rs1541254, and rs1541255, revealed five haplotypes demonstrating various frequencies: 0.65 for haplotype (1) with the major alleles (rs11210734T-rs1541252C-rs1541253C-rs1541254G-rs1541255A), 0.33 for haplotype (2) with the minor alleles (rs11210734C-rs1541252T-rs1541253T-rs1541254C-rs1541255G), 0.015 for the haplotype (3) (rs11210734T-rs1541252C-rs1541253C-rs1541254C-rs1541255A), 0.002 for haplotype (4) (rs11210734C-rs1541252T-rs1541253T-rs1541254C-rs1541255A), and 0.002 for haplotype (5) (rs11210734C-rs1541252T-rs1541253T-rs1541254G-rs1541255A). We first investigated the relationship between SM and the two most frequent haplotypes (1 and 2). The haplotype (1) was found to be associated with an increased risk of developing SM (*p* = 0.005). Among SM subjects, 55.8% were homozygous for the susceptibility haplotype (1) and 34.5% were heterozygous, whereas only 31.6% of the control subjects carried this haplotype at homozygote state. Individuals homozygous for haplotype (1) with major alleles have a higher risk of developing SM (*p* = 0.002, OR = 2.52) compared to heterozygous and homozygous haplotypes for minor alleles. This association remained significant when including age as a covariate in the statistical model (*p* = 0.006) (Table 3). We further performed the haplotype analysis by including rs10751450, rs10751451, rs10751452, rs11240734, rs1541252, rs1541253, rs1541254, and rs1541255. Among SM individuals, 47.8% were homozygous and 27.4% were heterozygous for the haplotype (CCTTCCGA) while only 26.6% of the control subjects carried this haplotype in the homozygous state. We found that haplotype with major alleles (CCTTCCGA) at the homozygous state was associated with an increased risk of SM (*p* = 0.003, OR = 2.67), even when taking age as a covariate (*p* = 0.005) (Table 3).

### 2.6. Lower Promoter Activity and Higher Enhancer Activity Are Associated with the Risk Haplotype of Severe Malaria

**Figure 5:**
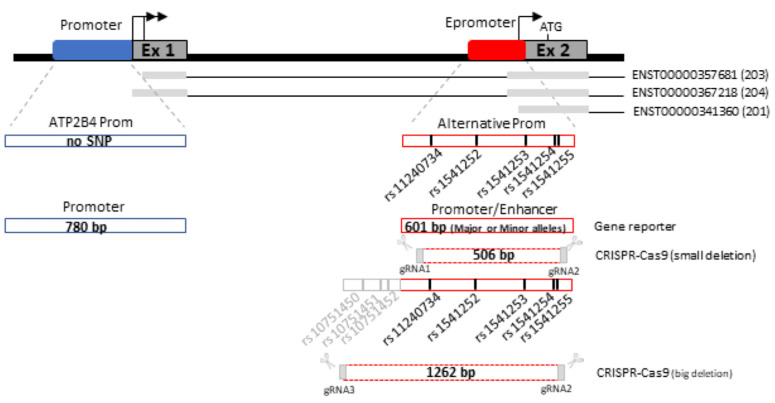
Description of the genomic regions used or deleted for luciferase gene reporter assay and CRISPR-Cas9 genome editing, respectively. The main *ATP2B4* promoter is illustrated in blue, whereas the alternative promoter containing the five SNPs studied is illustrated in red. For the luciferase gene reporter assay, the 601 bp genomic region was cloned first into the promoter position in pGL3-basic vector with the minor or major alleles, and then into the enhancer position in pGL3-Enhancer vector using the 780 bp genomic region as a promoter. For CRISPR-Cas9 editing a 506 bp fragment containing the five SNPs was deleted using gRNA1 and gRNA2. We also deleted a larger region containing eight SNPs (our five SNPs and three SNPs studied by Lessard et al.) using gRNA3 and gRNA2.

**Figure 6:**
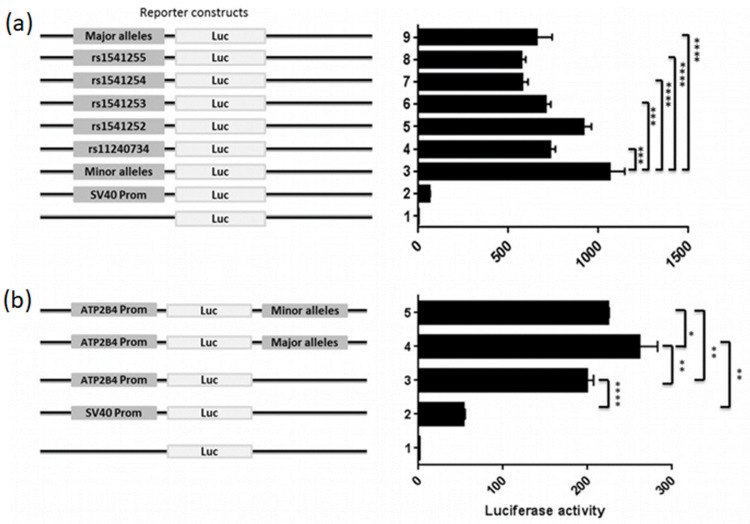
Luciferase reporter assay accessing the promoter and enhancer activity of *ATP2B4* variants in K562 cells. (**a**) Promoter activity of the DNA region containing *ATP2B4* Major and Minor haplotypes in addition to individual polymorphism. (**b**) Enhancer activity of *ATP2B4* SNPs with Major and Minor haplotypes. SV40 promoter and basic vectors were used as positive and negative controls, respectively. Data presented as the mean ± standard deviation of three independent experiments. **** *p* < 0.0001, *** *p* < 0.001, ** *p* < 0.01, * *p* < 0.05.

Luciferase reporter assays were performed in K562 cell lines to evaluate the regulatory effect of the 5 SNPs on *ATP2B4* expression. The candidate regulatory region and the position of the SNPs is portrayed in Figure 5. To assess whether this region may have a promoter function, we performed luciferase reporter gene assays by cloning it upstream the luciferase reporter gene. Interestingly, this regulatory region indicated strong promoter activity compared to the SV40 promoter for both the risk haplotype with major alleles (*p* < 1.10^−4^, 10.9-fold increase) and the non-risk haplotype with minor allele (*p* < 1.10^−4^, 17.7-fold increase) (Figure 6a). Moreover, the risk haplotype was shown to have reduced more transcriptional activity than the non-risk haplotype (*p* < 1.10^−4^). Hence, our results revealed differential allelic promoter activity in the K562 cell line, demonstrating that the non-risk haplotype exhibited a 1.6-fold increased transcriptional activity compared to the risk haplotype. In addition, we observed that each SNP has a specific impact on expression, and their combination reflects the combined effect observed. These results indicate that these five SNPs may be functional variants and that we must consider that it is their combined effect that is probably involved in the susceptibility to severe forms, as suggested by the haplotypes naturally present in individuals living in endemic areas.

To functionally confirm that this genomic region may have an enhancer function, we performed luciferase promoter assays by cloning it downstream of the luciferase reporter gene, whereas we cloned upstream a region of the main promoter of *ATP2B4* (Figure 5 and Figure 6b). We first validated the promoter activity of this 780-bp region compared to the SV40 promoter (*p* < 1.10^−4^, 3.8-fold increase). We further confirmed the enhancer activity of the 601 bp region containing the five SNPs on the main promoter of *ATP2B4* (*p* < 0.01, 1.3-fold increase for the construct with the 601 bp region as compared to the construct without this region, see Figure 6b). Moreover, we demonstrated that the region containing the major alleles exhibited an enhancer activity that was significantly higher than that of the region containing the minor alleles (*p* = 0.01). Also, there was a 1.2-fold increase in activity of the major allele haplotype relative to the minor allele haplotype. These data confirm that this regulatory region exhibits allele-dependent enhancer activity in the K562 cell line. Altogether, our results suggest that this regulatory region is a promoter with an enhancer function, also named Epromoter [31,32]. Furthermore, they suggest that genetic variants within this regulatory region orient the function of the region toward a promoter activity or toward an enhancer activity. 

### 2.7. Genome Editing Confirmed the Regulatory Activity of the Region Containing the SNPs

**Figure 7:**
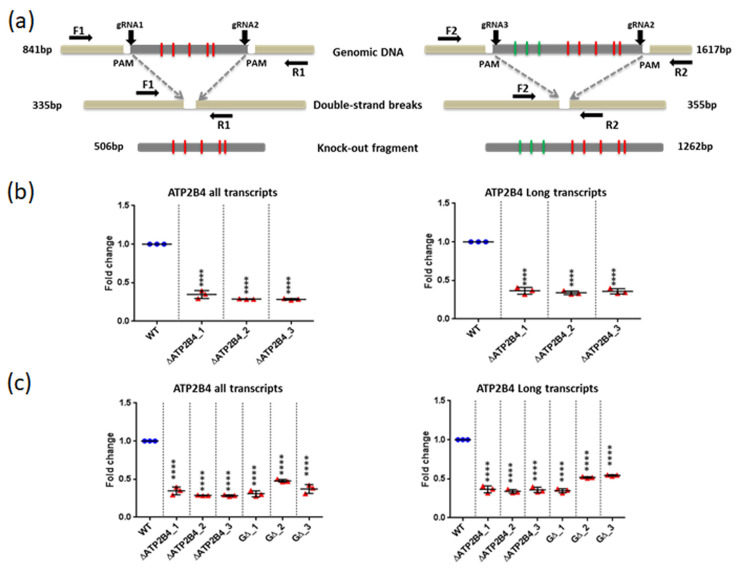
CRISPR-Cas9 mediated genome editing. (**a**) Principal strategy for the generation of DNA knockouts. gRNAs (gRNA1, gRNA2, and gRNA3) were designed flanking the genomic target to delete the two targeted DNA segments comprising either the five (506 bp) or the eight (1262 bp) candidate SNPs, creating double-strand breaks (DSBs) at 3 bp upstream of the PAM. The resulting DSB is repaired by the NHEJ pathway. The genomic deletion is detected by PCR using primers (F1, R1) and (F2, R2) providing the amplicons of 335 bp and 355 bp, respectively. (**b**) qPCR analysis of gene expression in wild-type K562 cells and the deleted *ATP2B4* clones (for the five candidate SNPs) to quantify either all the transcripts or only the two long transcripts of *ATP2B4*. (**c**) qPCR analysis of gene expression in wild-type K562 cells and the deleted *ATP2B4* clones (for the eight SNPs) to quantify either all the transcripts or only the two long transcripts of *ATP2B4*. Error bars illustrate standard deviation (*n* = 3 independent RNA/cDNA preparations): **** *p* < 0.0001.

To analyze the function of the Epromoter element, we deleted a DNA region of 506-bp containing the malaria-associated SNPs (rs11240734, rs1541252, rs1541253, rs1541254, and rs1541255) in the K562 cell line, using CRISPR/Cas9 mediated genome editing (Figure 5 and Figure 7a). We selected three deleted clones with the expected sequence and no additional edits. Expression analysis for total *ATP2B4* [including long and short transcripts (Figure 2)] demonstrated a 3.3-fold decrease in gene expression in deleted clones as compared to WT cells (*p* < 1.10^−4^) (Figure 7b). We also estimated the expression of the two long transcripts demonstrated in Figure 2 (*ATP2B4*-203 and *ATP2B4*-204) of *ATP2B4* (encoding for PMCA4a and PMCA4b) and we demonstrated a 2.8-fold decrease in deleted clones compared to WT cells (*p* < 1.10^−4^) (Figure 7b). These results support the hypothesis that this region has an enhancer function.

We deleted a region of 1262-bp to remove the eight SNPs (rs10751450, rs10751451, rs10751452, rs11240734, rs1541252, rs1541253, rs1541254, and rs1541255) (Figure 7a) and selected three clones validated by sequencing. Clones with this large 1262-bp deletion had a similar reduction in expression compared to the small 506-bp deletion, both for total expression and for long transcripts of *ATP2B4,* suggesting that the minimal region of 506 bp including rs11240734, rs1541252, rs1541253, rs1541254, and rs1541255 is enough to regulate the expression of *ATP2B4* (Figure 7c).

### 2.8. K562 Deleted Clones Exhibited Higher Intracellular Calcium Concentration

**Figure 8:**
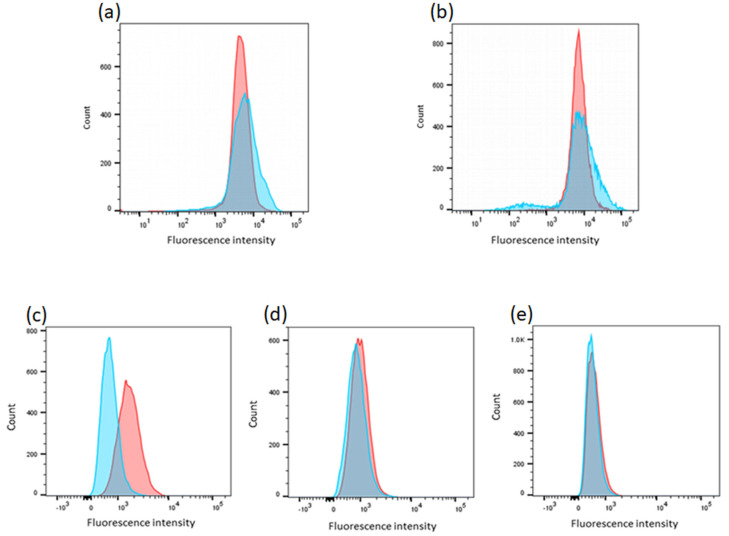
Visualization of calcium concentration and PMCA4 protein expression of K562 cells. (**a**,**b**) Visualization of calcium concentration of K562 WT (pink) and deleted cells (blue). An increased intracellular calcium concentration was displayed in clone ΔATP2B4_2 (**a**) and for clone ΔATP2B4_3 (**b**), respectively. (**c**–**e**) PMCA4 protein expression using flow cytometry analysis. Representative FACs plot illustrating isotype (blue) and JA9 (pink) peaks visualized by an APC conjugated secondary antibody in K562 WT cells (**c**), ΔATP2B4_2 deleted clone (**d**), and ΔATP2B4_3 deleted clone (**e**). These were visualized using FlowJo software.

Intracellular calcium concentration was measured in two clones (ΔATP2B4_2 and ΔATP2B4_3) deleted for the region containing rs11240734, rs1541252, rs1541253, rs1541254, and rs1541255 and a representative experiment is displayed in Figure 8 for each of the clones. Six and two experiments were performed with ΔATP2B4_2 and ΔATP2B4_3 clones, respectively. Supplementary Table S6 demonstrates descriptive statistics for each experiment performed with K562 wild-type (WT) cells and the two clones deleted for the genomic region of interest. First, we compared the mean of fluorescence intensity (MFI) of K562 WT cells with that of mutant clones, without considering the number of cells studied and the standard deviation (SD). The MFI of ΔATP2B4_2 and ΔATP2B4_3 clones was systematically higher than the MFI of K562 WT cells in each experiment (Supplementary Table S6). Non-parametric tests yielded significant differences when including only ΔATP2B4_2 clone results (*p* = 0.009) or including both ΔATP2B4_2 and ΔATP2B4_3 results (*p* = 0.029). Secondly, we tested whether K562 WT cells and the clones deleted for the genomic region of interest differed in their MFI, when considering the number of cells studied in each experiment and the corresponding standard deviation (SD). When analyzing each experiment, the wild-type cells and the clones deleted for the candidate region systematically differed in intracellular calcium concentration (*p* < 0.0001). The Cohen’s coefficient ranged from 0.14 to 0.56, demonstrating that the size effect was low. The statistical power was, however, close to 1 due to the large number of cells analyzed in each group (Table S6). Similarly, meta-analyses considering MFI, SD, and sample size provided evidence of an increase in calcium concentration in deleted clones when including only the results obtained with ΔATP2B4_2 clone (*p* < 0.0001) or including both ΔATP2B4_2 and ΔATP2B4_3 results (*p* < 0.0001). In all, the deletion of the region containing rs11240734, rs1541252, rs1541253, rs1541254, and rs1541255 was proven to cause a slight but significant increase in the intracellular calcium level.

### 2.9. PMCA4 Protein Is Not Expressed in K562 Deleted Clones

The expression of the protein PMCA4 has been assessed in the two deleted clones. Three independent experiments have been performed, indicating that the PMCA4 protein is not detected in the deleted clones (Figure 8), whereas this protein is expressed in K562 WT cells; the MFI difference between WT and deleted clones was significant (*p* = 0.002). Meta-analyses that considered MFI, SD, and sample size further provided evidence of a significant decrease of protein level in deleted clones (*p* < 0.0001). Overall, these results indicate that the deletion of the region containing rs11240734, rs1541252, rs1541253, rs1541254, and rs1541255 led to a dramatic fall in PMCA4 expression, a decrease of calcium efflux and an intracellular accumulation of calcium.

## 3. Discussion

Several GWAS have identified and replicated tagSNPs associated with SM. However, most of the causal variants remain unknown. Here we identified 5 *ATP2B4* regulatory SNPs associated with SM in a Senegalese population and in LD with the tagSNPs identified by GWAS (rs4951377 and rs10900585). We demonstrated that these five SNPs are in a promoter region that displays enhancer activity (Epromoter) [31,32].

Bioinformatic prioritization of the 125 non-coding SNPs in LD with the tagSNPs associated with SM indicated that the top 10 ranked SNPs by the IW scoring were on chromosome 1 within *ATP2B4*. When combining the IW scoring with the annotation of transcriptional regulator ChIP-seq peaks, rs1541252, rs1541253, rs1541254, and rs1541255 appeared to be the most promising candidates. Interestingly, these SNPs were close to rs11240734, ranked 1st and 5th based on the IW score and on the number of ChIP-seq peaks, respectively, suggesting that they may be located within a regulatory element. This hypothesis is also supported by (1) the co-location of the SNPs with epigenomic marks associated with promoter or enhancer activity, (2) the characterization of rs11240734, rs1541252, rs1541253, and rs1541254 as eQTLs in blood cells [33], (3) the association of rs1541252 and rs1541253 with PMCA4 levels [34], and (4) the identification of rs1541255 as a regulatory variant based on a reporter assay [28]. 

Consistently, the tagSNPs rs4951377 and rs10900585 ranked 51st and 61st using the IW score, and neither were in a region with histone marks or chip-seq peaks, suggesting that they are not the causal SNPs. Nevertheless, rs4951377 was an eQTL in blood cells [33], illustrating that an eQTL is not necessarily a regulatory variant. The association signal of rs4951377 with gene expression is likely explained by the effect of functional SNPs in LD with it. We confirmed that rs10751450, rs10751451, and rs10751452 ranked 3rd, 12th, and 13th, respectively, they could be regulatory SNPs as previously suggested [24], and they were located within an enhancer altering *ATP2B4* that codes for PMCA4 (Plasma membrane calcium transporting ATPase4) [24]. Noticeably, luciferase reporter assays provided evidence that these SNPs are regulatory variants [35]. This enhancer region is roughly 700 bp away from the region containing rs11240734, rs1541252, rs1541253, rs1541254, and rs1541255, which is potentially a regulatory region according to our results.

We confirmed the association between the tagSNP rs10900585 and SM in a Senegalese population with the same risk genotype and validated its association by meta-analysis, integrating all the SM populations [19,29,30]. In addition, we provided evidence of an association of the eight SNPs with SM in Senegal. Interestingly, these SNPs, except rs10751452, were imputed in the most recent GWAS of SM [17] and indicated a high Bayes Factor value (BF > 1.8 107) that was consistent with an association. Similarly, rs1541255 was associated with SM in Kenya [17], whereas the other SNPs were not genotyped in this population. 

**Figure 9:**
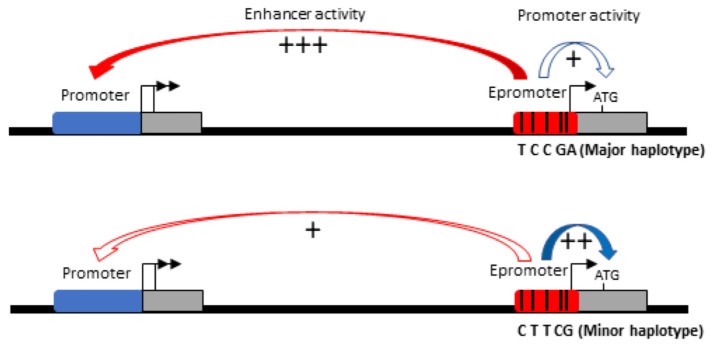
A model for transcriptional regulation of *ATP2B4* gene. The newly identified region containing the five SNPs studied (rs11240734, rs1541252, rs1541253, rs1541254, and rs1541255) corresponds to an alternative promoter with an enhancer function. Its activity is modulated by the combination of SNPs. The major haplotype had a stronger enhancer effect on the main *ATP2B4* promoter, increasing transcription of long transcripts, whereas the minor allele haplotype had stronger promoter activity, resulting in an increase in short transcripts.

Furthermore, we found a strong haplotype association with SM, and we observed that most of the Senegalese individuals exhibited either the haplotype combination of all the major alleles or the haplotype combination of all the minor alleles, whereas only a few individuals carried other haplotypes. The deletion of the region containing rs11240734, rs1541252, rs1541253, rs1541254, and rs1541255 strongly reduced the level of long transcripts. As this region is located within intron 1, this suggests that it acts as an enhancer on the main promoter, which controls the expression of the long transcripts (Figure 9). Luciferase gene reporter assays further indicated that this region increased the activity of the main promoter of *ATP2B4*.We demonstrated that this region has a promoter activity using luciferase reporter assays; this supports the hypothesis that it is also an alternative promoter (Figure 9). In addition, we observed higher enhancer activity with the haplotype combining the major alleles whereas this haplotype indicated lower promoter activity compared to the haplotype with the minor alleles. This result supports a hypothetic model of regulation proposed recently by Gao et al., Hua et al., and Ying et al. [36,37,38], whereby genetic variants can impose a regulatory switch between the enhancer and the promoter activity (Figure 9). It implies that major allele haplotype would increase the level of long transcripts, the expression of which is controlled by the main *ATP2B4* promoter, whereas minor allele haplotypes would increase the level of short transcripts, the expression of which is controlled by the alternative promoter containing the studied SNPs. It should be stressed, nevertheless, that mutagenesis or homologous recombination experiments in K562 cells are needed to confirm this hypothesis.

Our results further suggest that there was an effect of the combination of several SNP alleles on gene expression, thus reflecting the interaction between these SNPs. Although little is known about haplotypes’ influence on gene expression, Ying et al. [38] recently proposed a method to detect eQTL haplotypes and suggested that they are enriched in regulatory regions such as promoters or enhancers. Our finding is also supported by a study providing evidence at numerous loci that “multiple enhancer variants” cooperatively contribute to altered expression of their gene targets and that target transcript levels tend to be modest [39]. This raises the question of the natural combination of alleles in African populations under selective pressure, and supports our finding that the combination of these five SNPs was responsible for susceptibility to SM and that there is not a single causal variant. 

We also demonstrated that the deletion of this regulatory region containing rs11240734, rs1541252, rs1541253, rs1541254, and rs1541255 decreased both the global *ATP2B4* expression and specifically the expression of the long transcripts in the K562 cell line, suggesting that it is a promoter with an enhancer function (Epromoter). Consistently, decreasing expression of the transcripts in the deleted clone results in the absence of the PMCA4 protein and increased intracellular calcium. 

After deleting a region containing rs10751450, rs10751451, and rs10751452, Lessard et al. demonstrated a reduction in *ATP2B4* expression and a cytoplasmic calcium accumulation [24]. Hence, we included these SNPs in our study, and we obtained similar association results when adding rs10751450, rs10751451, and rs10751452 in the haplotype analysis, but also a similar decrease in *ATP2B4* expression for the clones either with five or eight SNP deletions. This finding supports that rs11240734, rs1541252, rs1541253, rs1541254, and rs1541255 affect the *ATP2B4* gene expression as much as rs10751450, rs10751451, and rs10751452 and contribute to the development of SM. Here, we have demonstrated the potential impact of these variants in regulating *ATP2B4* expression and intracellular calcium levels. We hypothesize that changes in calcium homeostasis may affect the growth of the parasite in red blood cells, and may also affect the clumping of infected red blood cells and their sequestration to the brain microvessels, thus contributing to SM susceptibility. In addition, our results support the hypothesis that a calcium-activated potassium channel may be activated, resulting in potassium efflux, dehydration, red blood cell volume loss, increased mean corpuscular hemoglobin concentration, and reduced parasitemia in malaria patients [24]. Indeed, rs1541252 and rs1541255 were found to be associated with mean corpuscular hemoglobin concentration in African-American children and parasitemia in malaria patients, respectively [29,40]. However, this hypothesis was recently challenged by Villegas-Mendez et al. and Pance et al. [41,42]. Pance et al. reported that *ATP2B4* deletion fully inhibited PMCA4 expression but had a very slight effect on the parasite growth in human stem cell-derived erythroid cells [42], whereas Villegas-Mendez et al. demonstrated that *ATP2B4* gene targeting did not alter parasitemia in mice infected by *Plasmodium berghei* but did protect mice by against cerebral malaria induced by *Plasmodium berghei* [41]. Interestingly, PMCA1 expression was increased in *ATP2B4*^−/−^ mice [41]. This suggests that PMCA1 expression and other calcium channels may compensate for a low expression of *ATP2B4* gene and thus PMCA4 protein in erythrocytes. Also, calcium concentration and parasite growth may not be dramatically altered by a strong inhibition of *ATP2B4* gene expression. 

Conversely, the *ATP2B4* gene is expressed in multiple cell types, and it is not excluded that the modulation of intracellular Ca^2+^ concentration alters the physiology of other cell types. For example, the regulation of intracellular Ca^2+^ signaling is a major determinant of CD8+ T cell responsiveness, which may have an important role in determining SM. Moreover, the PMCA4 protein encoded by *ATP2B4* is expressed both in the red blood cell and the brain with PMCA4b and PMCA4a the most abundant isoforms in red blood cells and in the brain, respectively [43]. Because these isoforms are tightly and specifically regulated in tissues and cells, we can therefore propose that dysregulation of calcium homeostasis in the brain could also be directly involved in cerebral malaria susceptibility as for different brain disorders such as Alzheimer’s disease and Parkinson’s disease. PMCA4a, which is the major isoform present on endothelial cells, may also play a crucial role in SM through its role as a negative regulator of vascular endothelial growth factor (VEGF)-activated angiogenesis. In cerebral malaria, the binding of parasitized erythrocytes to the cerebral endothelium and the consequent angiogenic dysregulation play a key role in pathogenesis [44]. VEGF, a regulator of endothelial inflammation and integrity, is involved in modulating tissue pathology in response to *P. berghei* infection. Indeed, a potent inhibitor to the VEGF signaling pathway dramatically aggravated the course of *P. berghei* infection [45].

## 4. Materials and Methods 

### 4.1. Study Subjects, Blood Samples, and Phenotypes

Malaria patients were recruited from two major Senegalese sites, the principal hospital of Dakar and the regional hospital of Tambacounda, including 90 cerebral malaria cases and 27 severe noncerebral cases, as described [46]. The control samples (*n* = 79) were obtained from healthy volunteers living in Dakar. Written informed consent was obtained for each patient and their accompanying family members. The study was approved by the institutional research ethics committee of the Université Cheikh Anta Diop. 

Venous blood samples and biological data including parasite density, hematology, and other characteristics were collected on the day of admission. The presence of *Plasmodium falciparum* was determined by at least two trained biologists by microscope examination of thin and thick smears before antimalarial treatment. If asexual parasites were observed, then the slide was considered as positive, and the number of parasitized erythrocytes was counted per µL of blood.

### 4.2. Bioinformatic Prioritization and Functional Annotation of Genetic Variants 

Significant GWAS signals replicated at least in an independent population were identified and the corresponding tagSNPs (rs4951377, rs10900585, rs186873296, rs62418762, and rs8176719) were selected for further analysis. Haploreg v4.1, a tool including LD information from the 1000 Genome project, was used to identify the SNPs in LD with these tagSNPs. This analysis of the African population and LD threshold r^2^ > 0.6 resulted in the selection of 125 SNPs. To prioritize the SNPs with putative functional significance, we performed bioinformatic analysis using the IW scoring annotation tool (https://www.snp-nexus.org/IW-Scoring/, accessed on 6 March 2019) [25] and ReMap 2018 based on DNA-binding ChIP-seq experiments [26]. We also checked whether these SNPs have been annotated as eQTLs (https://pubs.broadinstitute.org/mammals/haploreg/haploreg.php, accessed on 1 March 2019) [27] or identified as regulatory SNPs using the massive parallel reporter assay named Sure-seq [28]. Additional analysis has been performed to evaluate whether the candidate SNPs are located within a DNAse I hypersensitivity region, peaks of H3K4me1, H3K4me3, and H3K27ac marks in the K562 cell line, which were visualized in the UCSC genome browser.

### 4.3. DNA Extraction, DNA Amplification, and Genotyping

Genomic DNA was extracted and amplified as previously described [46]. In total, we genotyped nine SNPs of *ATP2B4* including rs11240734, rs1541252, rs1541253, rs1541254, and rs1541255, the TaqSNP rs10900585, and the SNPs rs10751450, rs10751451, and rs10751452, previously described as regulatory variants [24]. Genotyping was performed using either the Sanger sequencing or the TaqMan SNP genotyping assays (Life Technologies, Waltham, MA, USA). All the primer pairs for sequencing were designed using Primer 3 software. PCR amplification of DNA fragments containing rs11240734, rs1541252, rs1541253, rs1541254, and rs1541255 was performed with forward (5′-TCAGGCCTAGCTATCAGTTCAG-3′) and reverse (5′-CGAGTAGCCGTCCGAAGTC-3′) primers, respectively. For the tagSNP rs10900585, the forward and the reverse primers were (5′-GGGATGAGGAGGCTTACAGG-3′) and (5′-GAGGTTGAGGTGAGCGGATC-3′), respectively.

PCR amplification was executed in 50µL reaction volume with 12.5 ng of genomic DNA, GoTaq G2 2X ready-to-use Master Mix (Promega, cat#M7433), and 10 µM of each primer. For rs10900585, the annealing temperature was 65 °C, while for rs1541252, annealing was at 60 °C. The PCR products were submitted to electrophoresis on 2% agarose gel to verify the product size, and then purified with a PCR Purification kit (Qiagen, Hilden, Germany) following the manufacturer’s protocol. Five µL of purified PCR product was sequenced using the Sanger method (GATC biotech, Ebersberg, Germany, or Eurofins Genomics, Ebersberg, Germany). 

For rs10751450, rs10751451, and rs10751452, we used TaqMan genotyping assays C_31796478_10, C_31796479_10, and C_31796480_10, respectively. PCR amplification was performed with the QuantStudio 6 Flex Real-Time PCR Systems (Life Technologies, Waltham, MA, USA) in a final volume of 5 µL containing 2.1 µL of Master Mix (Life Technologies, Waltham, MA, USA), 0.06 µL of TaqMan probe, 1.84 µL H_2_O, and 1 µL of DNA (10–15 ng/µL).

### 4.4. Luciferase Gene Reporter Assay and Site-Directed Mutagenesis:

#### 4.4.1. Promoter Activity

A 601bp DNA fragment upstream from the *ATP2B4* translation start site (chromosome 1: 203682549–203683049 according to the hg38 assembly) was cloned into the MlulI-XhoI sites of the pGL3-basic vector (Promega, Madison, WI, USA), which contained the firefly luciferase coding sequence (GeneCust, Boynes, France). We obtained two different pGL3 constructs containing either minor or major alleles of the five SNPs (rs11240734, rs1541252, rs1541253, rs1541254, and rs1541255). From the minor allele construct, we generated additional constructs by replacing the minor allele with the major allele of each SNP individually. The site-directed mutagenesis was performed using the Q5 Site-Directed Mutagenesis Kit (New England Biolabs, Ipswich, MA, USA,) and primers were designed by NEBaseChanger tool and provided by the supplier (detail sequences provided in Supplementary Table S7). K562 cells (ATTC CLL-243) were grown in Gibco RPMI 1640 medium (Thermo Fisher Scientific, Waltham, MA, USA), supplemented with 10% FBS (fetal bovine serum). K562 transfection was performed with the Neon^TM^ Transfection system (Invitrogen) according to the manufacturer’s instructions. For each of the nine tests performed, 10^6^ cells were co-transfected with 1 µg of control vectors: (1) negative control vector (empty pGL3-basic vector (cat# E1751)) or (2) positive control vector (pGL3-promoter vector (cat# E1761)), or with 1 µg of the construct to be tested (a pGL3-basic vector containing either the minor alleles CTTCG or the major alleles TCCGA or one of these combinations: TTTCG, CCTCG, CTCCG, CTTGG, or CTTCA) and 200 ng of pRL-SV40 (a plasmid encoding renilla luciferase from Promega (cat# E2231)), which was used as a transfection efficiency control. Transfected cells were maintained at 37 °C in 5% CO_2_ for 24 h. Values of firefly and renilla luciferase were obtained by analyzing 20 µL of cell lysate according to the standard instructions provided in the Dual-Luciferase kit (Promega, Madison, WI, USA) in a TriStar LB 941 Multimode Microplate Reader (Berthold Technologies, Thermo Fisher Scientific, Waltham, MA, USA). Firefly luciferase activity of each sample was normalized to renilla luciferase and expressed as the fold change of the empty vector control. 

#### 4.4.2. Enhancer Activity

A 780-bp fragment of *ATP2B4* promoter (chromosome1: 203626081-203626860 (hg38 assembly)) was cloned into pGL3-Enhancer vector (Promega, Madison, WI, USA) between MluI- Xho I sites and the 601 bp fragment of *ATP2B4* enhancer (chromosome 1: 203682549-203683049 according to the hg38 assembly) containing respective minor and major alleles of the five SNPs was cloned into BamHI-SalI sites. Luciferase assays were performed in K562 cells as described above. 

### 4.5. CRISPR-Cas9 Genome Editing in K562 Cells

Using the CRISPR design tool provided by IDT, we identified guide RNAs to target the *ATP2B4*-specific region. Chemically synthesized oligoribonucleotides were manufactured by IDT: the crRNAs (35 mer with specific part to DNA target sequence) and the universal tracrRNAs (67 mer). A two-part system where synthetic crRNA (5 µL of 100 µM) and tracrRNA (5 µL of 100 µM) were annealed to form an active gRNA complex. Cas9 RNP complexes were assembled in vitro by incubating 3.4 µL Cas9 protein (62 mM) with 4.8 µL active gRNA complex (cr:tracrRNA) and 1.8 µL of PBS. To generate the genomic deletion, two different RNP complexes were simultaneously electroporated with the Neon transfection system into 5.10^5^ K562 cells. The small deletion of 506 bp (containing the five SNPs) was obtained by using 2 µL gRNA1 and 2 µL of gRNA2 (details of sequences in Supplementary Table S8). The large deletion of 1262 bp (containing the 8 SNPs) was performed by using gRNA2 and gRNA3 (Supplementary Table S8). Those bulk cultures transfected with tandem gRNA were plated clonally in 96-well plates at a limiting dilution less than 0.5 to avoid mixed clones. After approximately 14 days of clonal expansion, amplification of DNA was performed directly from the clones by using the 2X Phire Tissue Direct PCR Master Mix containing Phire Hot Start II DNA Polymerase (Thermo Fisher Scientific, Waltham, MA, USA), with 20 µL of dilution buffer and 0.5 µL of DNA Release additive, which allowed improved release of DNA from the cells. Clones were screened for deletion by PCR using primers F1 and R1 for the small deletion detection, which would produce a specific short fragment of 335 bp in the presence of deletion and a long fragment of 841 bp in the absence of deletion (Supplementary Table S8). Primers F2 and R2 were used for large deletion detection that would produce amplicons of 355 bp in presence of deletion and 1617 bp in the absence of deletion (Supplementary Table S8). PCR was performed using 10 µL of 2XMaster Mix and 10 µM of each primer, annealing at 60 °C. After electrophoresis on 1% agarose gel, we selected homozygote deleted clones displaying only amplicons of 335 bp or 355 bp for the small and the large deletions, respectively. After sequencing using the Sanger method, only the clones with the appropriate sequence with no additional edits were kept for gene expression analysis.

### 4.6. Reverse Transcription-Quantitative PCR

Total RNA was extracted for each selected clone and wild-type K562 cell using the RNeasy mini kit (Qiagen, Hilden, Germany). One µg of RNA per clone was converted into cDNA using Superscript VILO Master Mix (Invitrogen, Thermo Fisher Scientific, Waltham, MA, USA). Three independent RNA/cDNA preparations were performed for each clone. Real-time quantitative PCR (RT-qPCR) was subsequently performed using SYBR Select Master Mix (Thermo Fisher Scientific, Waltham, MA, USA) on a QuantStudio 6 Flex instrument. Primers were designed using Primer 3 software to span exon 1 of *ATP2B4* (F3 and R3) to specifically quantify the long transcripts (ENST00000357681, ENST00000367218) or to span exon 19 and exon 20 (F4 and R4) to quantify all the *ATP2B4* transcripts (ENST00000357681, ENST00000367218, ENST00000341360, ENST00000458092, and ENST00000356729) (Supplementary Table S9). PCR efficiency was validated by performing serial dilution analysis. Gene expression was normalized to that of actin and the relative expression was calculated by the ∆CT method. The data reported correspond to the mean of triplicates from three independent experiments per clone and expressed as fold change relative to wild-type cells. Three independently generated clones were analyzed for the gene expression quantification of *ATP2B4*.

### 4.7. Calcium Measurement by Flow Cytometry in K562 Clones and Wild-Type Cells 

The intracellular quantity of calcium was measured in K562 cells and ΔATP2B4_2 and ΔATP2B4_3 clones by flow cytometry using the Fluo-4 AM kit (Molecular Probes/Thermo Fisher Scientific, Waltham, MA, USA), following manufacturer’s instructions. Briefly, samples of 10^6^ cells were harvested for each cell type. The cells were pelleted and resuspended in 1 mL of culture medium without serum. One ml of either Fluo-4-AM (labeled cells) or kit buffer (control cells) was added to the cells, before 1 h incubation at 37 °C. The cells were then directly observed on an LSRFortessa X-20 cytometer (BD Biosciences, NJ, NJ, USA). Cells were gated on forward/side-light scatter and the fluorescence intensity of 20,000 cells was measured at the FITC excitation/emission wavelength for each sample.

### 4.8. Detection of PMCA4 by Flow Cytometry in K562 Clones and Wild-Type Cells

The presence of PMCA4 in K562 cells and clones ΔATP2B4_2 and ΔATP2B4_3 was detected by flow cytometry, using the specific antibody JA9 (ABCAM, ab2783). The labeling was realized according to a slight modification of the manufacturer’s protocol. For each cell type, 2 × 10^6^ cells were harvested, pelleted, and fixed in 2 mL of 80% methanol (5 min). After centrifugation, the cells were incubated in 2 mL of PBS with 1% BSA for 30 min at room temperature to block nonspecific interactions. Half of each cell type was then incubated with the JA9 antibody (2 µg/1 × 10^6^ cells) for 30 min at room temperature. The other half was used for isotypic control (ab170190, 2 µg/1 × 10^6^ cells). After centrifugation, the cells were incubated with the secondary antibody (goat anti-mouse IgG1 APC, ab 130786) at 1/200 dilution in PBS with 1% BSA, for 30 min at room temperature. Finally, cells were washed twice in PBS and observed on an LSR Fortessa X-20 cytometer (BD Biosciences). Cells were gated on forward/side-light scatter and the fluorescence intensity of 20 000 cells was measured at the APC excitation/emission wavelength for each sample.

### 4.9. Statistical Analysis

A Chi-squared test was used to determine whether the genotype distribution in healthy Senegalese subjects conformed to the Hardy–Weinberg equilibrium. Chi-squared tests and logistic regression analyses were carried out with SPSS (statistical software version 20) to assess the association between the SNPs and SM in Senegalese subjects. Differences were considered significant if the P-value obtained in a two-tailed test was <0.05. The odds ratio (OR), 95% confidence intervals (CIs), and the influence of covariates on the phenotype were evaluated by logistic regression analysis. A meta-analysis of the genetic association was performed using MetaGenyo [47]. Statistical differences between luciferase constructs or between clones were performed using Student’s t-tests. Mixed models were also used to consider the triplicates performed for each independent experiment. *P* values < 0.05 were considered statistically significant. Plot generation was performed using GraphPad Prism. Statistical analysis to evaluate the difference in the calcium concentration between deleted and wild-type clones was based on the non-parametric Wilcoxon test and the t-test using SPSS, and on a meta-analysis approach using Open Meta-Analyst software [48]. All the tests used were two-sided tests.

## 5. Conclusions

We identified a new regulatory region that controls the expression of both *ATP2B4* mRNA and PMCA4 proteins, and that affects intracellular calcium levels. We also demonstrated that the activity of this regulatory region was perturbed by five genetic variants associated with SM. This suggests that we identified causal variants within a locus identified through GWAS of SM. This also fosters the development of therapeutic strategies based on the modulation of *ATP2B4* expression and calcium levels. It should be stressed, however, that the effect size of *ATP2B4* was modest in our study population, consistent with previous heritability studies [4,17,29]. More generally, genetic variants in *ATP2B4*, *FREM3-GYP A/B*, *EPHA7*, *ABO*, and *HBB*, which have been identified by several GWAS studies, explain 11% of the genetic contribution to variation in SM susceptibility [17]. In this way, a polygenic effect has been proposed by Damena et al. (2020), suggesting that many genetic variants with a small effect size remain to be discovered [4].

## Figures and Tables

**Table 1:** Numbers of transcription factor ChIP seq peaks and IW scoring rank for the promising candidates.

SNP rsID	Genomic Coordinate (hg38)	IW Scoring Rank	Number of ChIP-seq Peaks	eQTL Hits
rs11240734	203682695	1	54	3 hits
rs1541252	203682798	2	90	3 hits
rs10751450	203681816	3	46	
rs2228445	203698280	4	18	3 hits
rs202111522	203682602	5	43	
rs1541254	203683011	6	104	
rs1541255	203683012	7	103	
rs10594838	203685540	8	2	
rs10625220	203686622	9	15	
rs1541253	203682911	10	149	
rs8176719	133257721	11	7	14 hits
rs10751451	203681849	12	49	3 hits
rs10751452	203681901	13	50	

**Table 2:** Association of *ATP2B4* SNPs with severe malaria in the Senegalese population-based study.

SNP	Position ^a^	Minor Allele	MAF ^b^	Risk Genotype	Controls%	Severe Malaria %	OR	95% CI	*p*
rs10900585 (T > G)	203684896	G	0.41	TT	32.9	48.7	1.94	1.07–3.50	0.029
rs11240734 (T > C)	203682696	C	0.40	TT	31.6	53.8	2.52	1.39–4.58	0.002
rs1541252 (C > T)	203682799	T	0.40	CC	31.6	53.8	2.52	1.39–4.58	0.002
rs1541253 (C > T)	203682912	T	0.40	CC	31.6	53.8	2.52	1.39–4.58	0.002
rs1541254 (G > C)	203683012	C	0.43	GG	31.6	53.8	2.52	1.39–4.58	0.002
rs1541255 (A > G)	203683013	G	0.40	AA	31.6	53.8	2.52	1.39–4.58	0.002
rs10751450 (C > T)	203681817	T	0.41	CC	32.9	50.4	2.07	1.15–3.75	0.016
rs10751451 (C > T)	203681850	T	0.40	CC	34.2	56.4	2.49	1.38–4.50	0.002
rs10751452 (T > C)	203681902	C	0.39	TT	35.4	55.6	2.27	1.26–4.09	0.006

An association study has been performed in the Senegalese population including 117 severe malaria cases and 79 healthy controls. *p*-values and OR were calculated according to a logistic regression test without any covariate. ^a^ Data represent the position on chromosome 1 according to human hg38 coordinates. ^b^ MAF (Minor allele frequency) was estimated from Senegalese healthy controls. OR, odds ratio; CI, confidence interval.

**Table 3:** Haplotype association analysis for the five and eight SNPs in the Senegalese population.

			SM vs. Ctrl		
Major Haplotype	Minor Haplotype	Risk Haplotype	*p*-Value	OR	95% CI
TCCGA	CTTCG	TCCGA/TCCGA	0.002 ^a^	2.52 ^a^	1.39–4.58 ^a^
			0.006 ^b^	2.38 ^b^	1.29–4.40 ^b^
CCTTCCGA	TTCCTTCG	CCTTCCGA/CCTTCCGA	0.003 ^a^	2.67 ^a^	1.41–5.04 ^a^
			0.005 ^b^	2.53 ^b^	1.32–4.86 ^b^

Haplotype analysis has been performed on the two most frequent haplotypes including severe malaria cases and healthy controls. *p*-values and OR were calculated according to logistic regression. ^a^ The statistical model compares individuals homozygous for major allele haplotypes with individuals heterozygous or homozygous for minor haplotypes without any covariate. ^b^ The statistical model considers age as a covariate. OR, odds ratio; CI, confidence interval.

## Data Availability

The datasets used and/or analyzed during the current study are available from the corresponding author on reasonable request.

## Supplementary Materials

The following are available online at https://www.mdpi.com/article/10.3390/ijms23094849/s1.
