# Supplementary figures and images for: Identification of ATP2B4 Regulatory Element Containing Functional Genetic Variants Associated with Severe Malaria
Source: Int J Mol Sci. 2022 Apr 27;23(9):4849. doi: 10.3390/ijms23094849 (PMC9101746; doi:10.3390/ijms23094849)

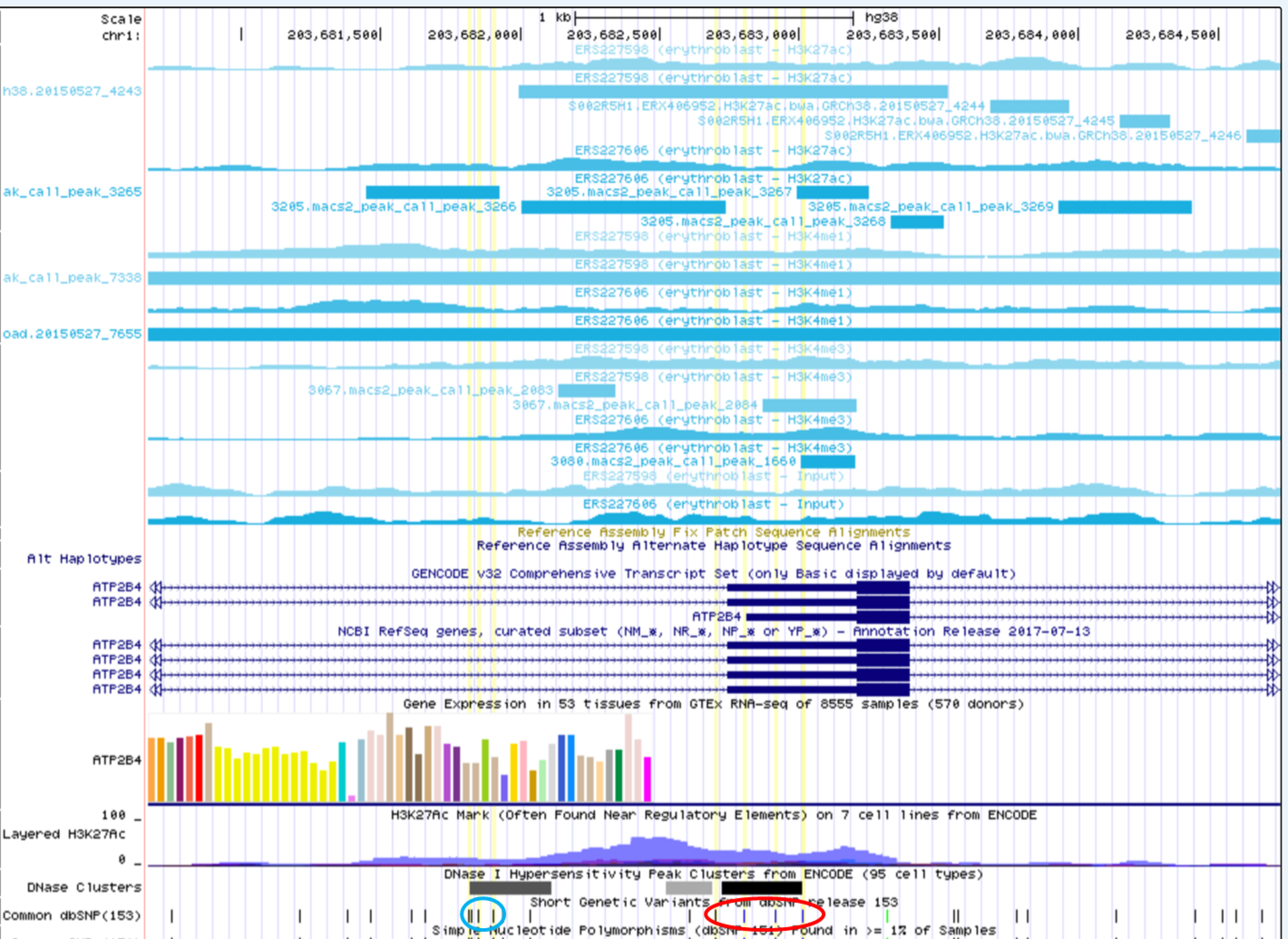

Supplement: Supplementary file 1 [file ijms-23-04849-s001.zip › Supplementary figure S1.tif]
